# Biological Properties and Phytochemicals of Multipurpose Tree Plant *Hagenia abyssinica*

**DOI:** 10.3390/molecules29245871

**Published:** 2024-12-12

**Authors:** Varun Jaiswal, Hae-Jeung Lee

**Affiliations:** 1 Department of Food and Nutrition, College of BioNano Technology, Gachon University, 1342 Seongnam-daero, Sujeong-gu, Seongnam-si 13120, Republic of Korea; computationalvarun@gmail.com; 2 Institute for Aging and Clinical Nutrition Research, Gachon University, Seongnam-si 13120, Republic of Korea; 3 Department of Health Sciences and Technology, Gachon Advanced Institute for Health Sciences and Technology (GAIHST), Gachon University, Incheon 21999, Republic of Korea

**Keywords:** *Hagenia abyssinica*, phytochemicals, therapeutics, cancer, diabetes, toxicity, antioxidants, anti-inflammatory

## Abstract

*Hagenia abyssinica* (HA) is a monotypic tree species used as traditional medicine against various diseases and conditions in African countries. HA is also a multipurpose plant used for furniture, fuel wood, soil fertility management, and rainwater conservation, along with medicinal usage. In different in vitro, in vivo, ex vivo, and human studies, the potential of HA for different pharmacological properties, including anti-parasite, antibacterial, antifungal, antispasmodic, anticancer, anti-diabetes, antidiarrheal, wound healing, antioxidant, and anti-inflammatory activities were observed. Antioxidant and anti-inflammation properties of HA may be the primary reason for the multi-pharmacological activities of HA. Initial toxicity studies and the presence of various phytochemicals, especially flavonoids, also support the therapeutic potential of HA. The diverse medicinal properties of the plant have different challenges to overcome for its development. Limited studies to decipher the molecular mechanism behind the pharmacological activity restrict the utilization of the complete potential of HA as therapeutics. Still, the compilation of phytochemical, pharmacological activities, and target pathways of HA is missing in the literature. The current review not only compiles the pharmacological activities and phytochemicals but also highlights the gaps and proposes the future direction to develop HA as a candidate against important diseases.

## 1. Introduction

Since the prehistoric era, flora has been used as an important source of remedies for the prevention and treatment of various diseases. In different parts of the world, the local population largely depends on the regional plants for health needs. *Hagenia abyssinica* (Bruce) J.F. Gmel is an important medicinal plant that supports some of the primary medical needs of the local population from generations in different African countries, especially in Ethiopia and Kenya [1]. *Hagenia abyssinica* (HA) is a tree plant commonly known as Kosso and also called African redwood.

In 1645, a Portuguese priest, Godinho, described HA as an anthelmintic medicine used by Ethiopian people, which was added to European pharmacopeia in the last century. Various medicinal uses of HA in traditional medicine in the resident population include the treatment of intestinal worms, diarrhea, abdominal pain, sexually transmitted diseases, typhoid, hepatitis, cancer, epilepsy, cold, cough, stomach pain, contraceptives, wound healing, and Livestock disease [1,2]. The main part of the plant used for medicine is reported to be the female flowers. However, leaf, root, bark, and wood are also used for medicinal purposes [1]. The study also indicated some toxicity of HA at high doses in animal models which suggested that some caution may be required during the therapeutic use of HA [3].

Apart from its medicinal use in humans and livestock, HA is used for construction, furniture, fuel wood, soil fertility management, and rainwater conservation, making HA a multipurpose plant [4]. Earlier, HA was the most abundant species in the semi-humid mountain woodlands of Ethiopia. The overexploitation due to the various uses and climate change posed a serious threat to HA in the natural environment that demands the sustainable use of the plant. In view of the threat and importance of the plant, the in vitro micropropagation protocol has also been developed for HA [4].

Considering the importance of HA in traditional medicine, pharmacological studies have been conducted that highlight the several potential applications of HA against parasite worms, bacterial infection, cancer, diabetes, diarrhea, and wound healing. Antioxidant and anti-inflammatory activities of HA were also reported in different studies [2,5,6], which may explain the numerous pharmacological activities of HA as these activities may contribute positively against different diseases and conditions [7,8].

Additionally, important types of phytochemicals known for imperative pharmacological importance such as alkaloids, polyphenols (tannins, flavonoids, and anthraquinones), phenol derivatives (such as phloroglucinol derivatives), and terpenoids (saponins and steroids) were also identified from HA, which may explicate the different medicinal properties of HA [9,10].

Despite the numerous potential health benefits observed in several studies and the presence of important phytochemicals, a compilation of the pharmacological properties and phytochemicals of HA is still not available. Thus, the current study is conducted, not only to provide a comprehensive compilation of pharmacological properties and phytochemicals but also to highlight the gaps and shed light on further directions for the development of HA as herbal therapeutics against important diseases.

## 2. Electronic Literature Search Strategy

A literature search was carried out through several important literature databases such as ResearchGate, Scopus, and Web of Sciences. Keywords and their combination related to the study used in the literature search were *Hagenia*, *Hagenia abyssinica*, kosso, pharmacological activities, anti-cancer, anti-parasite, anti-inflammatory, anti-diabetes, etc. Relevant documents associated with the topic, such as research articles, review articles, and books and book chapters from the search results were included in the study.

## 3. Botanical Description

HA is a monotypic tree species of the Rosaceae family that is dioecious in nature. HA is found in Ethiopia, Kenya, Sudan, Uganda, Rwanda, Burundi, the Democratic Republic of Congo, Tanzania, Malawi, and Zambia. The Afromontane forests of East Africa are a well-suited location for HA. HA prefers the altitudinal range of 2400 to 3500 m, with annual rainfall of 1000–1500 mm. The size of the tree can attain a height of up to 20 m. HA is an anemogamous and anemochorous tree plant with distinct male and female flowers [11]. HA has wide imparipinnate leaves sized up to 40 cm long with five to eight pairs of major leaflets, alternating with a few small, sub-circular leaflets. The several flowers are arranged in paniculate inflorescence, which is up to 60 cm long. The male and female flowers are different and typically present on distinct plants. Male flowers are small (~0.8 cm diameter) and white to orange in color, while the female flowers are bigger (1.5 cm diameter) and red in color. In the flower, the epicalyx is present, with four to five sepals and petals, while up to 20 stamens may be present. The fruits of HA are achene, enclosed within the calyx tube (https://www.treesandshrubsonline.org/articles/hagenia/hagenia-abyssinica/, accessed on 10 October 2024).

## 4. Toxicity Studies

Multiple potential pharmacological activities of HA have encouraged different types of toxicity studies for HA. In the toxicity study on Vero cell lines, the hexane and methane extracts of stem bark were found to be non-toxic (CC_50_ > 100 μg/mL). In acute toxicity studies on mice, the leaf extract of HA was found to be safe up to 5000 mg/kg body weight per day [12]. The different organic extracts of leaves and stem barks of HA did not show any significant toxicity in mice. However, at high doses of 5000 mg/kg water extract, 20% mortality was observed in the mice [12]. The dermal toxicity of flower extract was studied before its potential application in wound healing. Methanol extract of flower (10% *w*/*w*) was used and applied for 24 h, and it was found to be safe in 14 days of monitoring in the mice. No sign of toxicity, erythema, edema, or mortality was reported during the observation periods [6]. In an acute oral toxicity test, a 2000 mg/kg dose was found to be safe in the study. Similarly, methanolic leaf extract of HA was also studied for oral acute toxicity. Like the previous study, the leaf extract at 2000 mg/kg dose was found to be safe in 14 days of monitoring in the female albino mice [13]. The toxicity study for intraperitoneal administration of aqueous and hydro-alcoholic extracts on the adult albino mice revealed the LD_50_ of flowers were 2014 ± 301 and 1980 ± 179 mg/kg, respectively [14]. The retinotoxicity effect of female flowers of HA on Male Australorp-Leghorn cross chicks was also studied at high (0.25 g) and low (0.025 g) doses in a nine-day experiment. Dose-dependent reduction in visual discrimination for food grains was observed in the chicks with a total dose of 0.25 g. In the high dose (2.25 g), degeneration of ganglion cells was also observed in the anatomical study. However, no retinal lesions were observed in the birds that consumed a cumulative dose of less than 0.25 g [3].

## 5. Phytochemicals of HA

Initially, the motivation for the identification of phytochemicals from HA was the potential medicinal application and the possibility of toxicological manifestation of HA. The first substance produced by Merck Germany (Darmstadt, Germany) from HA (female flowers) was kosins in 1874. Later, in 1894, the toxic substance named kosotoxin was isolated from HA [15]. Kosins are the groups of compounds that are phloroglucinol derivatives bearing isobutyryl, isovaleryl, and 2-methylbutyryl sidechains and they are primarily reported from the female and male flowers of HA. Currently, more than 40 phytochemicals have been reported from HA, which may explain the pharmacological properties of HA.

Mainly, the flowers, roots, and leaves of HA have been reported to be used in traditional medicine; thus, phytochemicals from these parts were analyzed. Screening of the methanolic extract of the flowers was conducted for phytochemicals, which revealed the presence of important phytochemical classes such as polyphenols (tannins, flavonoids, and anthraquinones) and terpenoids (saponins and steroids) in the flowers of HA [16]. In order to estimate potentially bioactive compounds and optimize solvents used for the extraction, the total phenolic and flavonoid content of the extract of HA was studied in different solvents (ethyl acetate, dichloromethane, and n-hexane). Both total phenolic content (TPC) and total flavonoid content (TFC) were found to be highest in the ethyl acetate fraction of extracts, which were 57.193 ± 0.001 μg Gallic acid equivalent (GAE)/g and 365.091 ± 0.001 μg Rutin equivalent (RE)/g, respectively. Later, the antioxidant activity of the extract was in positive correlation with the total phenolic and flavonoid content of the extract, which suggests the role of these phytochemicals in biological activity. Female and male flowers of HA were analyzed for phenolic constituents as these flowers have been used in traditional medicine for different medicinal purposes. Kosins and phenolic acids were isolated and identified from the flowers using different methods of analytical chemistry, including thin layer chromatography (TLC), liquid chromatography (LC), ultraviolet (UV), infrared (IR), mass spectroscopy (MS), and nuclear magnetic resonance (NMR) spectroscopy [17]. Identified phenolic acids were protocatechuic acid, p-hydroxybenzoic acid, and vanillic acid. Similarly, the identified kosins were α-kosin, kosotoxin, and protokosin. These kosin compounds were reported to have anticancer activities in animal models and are considered unique compounds in HA as kosins are not often present in plants. Kosins are pharmacologically active compounds that require precaution if used at high doses due to potential toxicity. The essential oil component of the female flower of the plant was also analyzed for its chemical composition [18]. In the study, 20 different phytochemicals were observed in the yellow color oil obtained from the flowers; ledol was the main component, constituting more than 50% of the oil (Table 1). Ledol is also reported from other plants, such as *Rhododendron tomentosum* and *Eriocephalus africanus* [19,20]. Ledol is considered a pharmacologically active compound with expectorant and antitussive effects. The toxicity of ledol at large doses restricts its usage as it can act on the central nervous system and can cause seizures, cramps, paralysis, breathing problems, and even death [19,20]. The high doses of extract HA may have large amounts of ledol and thus, the high doses of HA must be used with precautions.

To identify potential antioxidant and antiproliferative compounds, the ethyl acetate fraction of roots was subjected to high-performance liquid chromatography (HPLC)-UV/electrospray ionization (ESI)-MS/MS analysis, and specific compounds were isolated and identified. Nine phytochemicals (acacetin, caffeic acid, dehydrodicatechin A, dihydroquercetin, isoquercitin, quercetin, trans-ferulic acid, 2-methoxyterephthalic acid, and 3,4-dihydroxybenzoic acid) were identified through NMR and other spectroscopic methods [2] (Table 1). Although the anti-parasitic activity of HA has been utilized by the local population and supported through different in vitro studies, the knowledge of the phytochemical components important for anti-parasitic activities was limited. A recent study has been conducted to decipher the phytochemicals behind the anti-parasitic activity of the plant through Ultrafiltration-liquid chromatography–mass spectrometry (UF-LC–MS). Seven potential phytochemicals (protocatechuic acid, corilagin, brevifolin carboxylic acid, brevifolin, methyl brevifolin carboxylate, quercetin, and methyl ellagic acid) were identified through activity-linked identification and further analyzed through molecular docking against target enzymes with positive control molecules [2].

Preliminary screening of the methanolic extract of the leaves was also conducted for phytochemicals before anti-diabetes activity, which revealed the presence of important phytochemical classes such as polyphenols (flavonoids and anthraquinones) and terpenoids (saponins) in the leaves of HA [23].

## 6. Pharmacological Activities of HA

**Figure 1:**
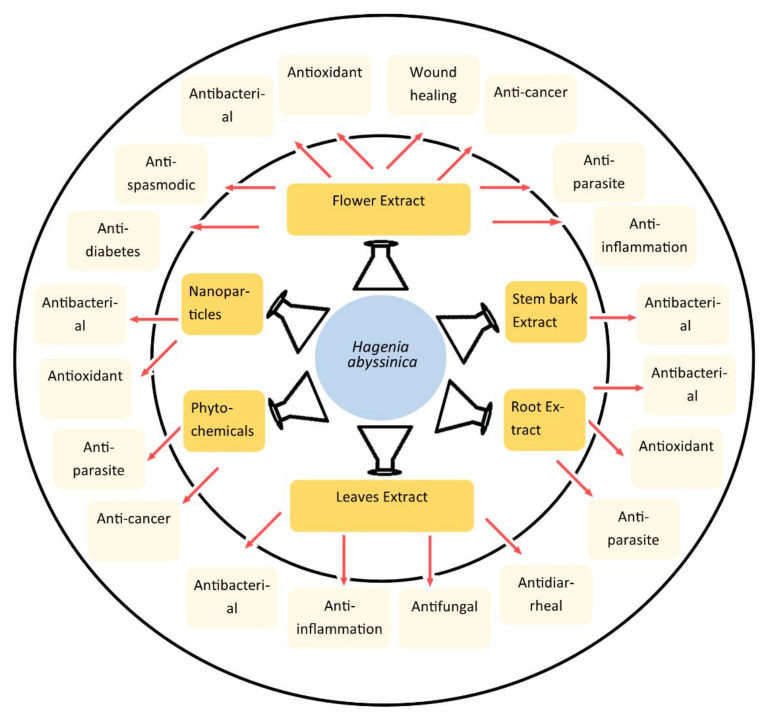
The pharmacological activities of different parts of *Hagenia abyssinica* were studied through various types of extracts.

The medicinal properties of HA were described from the traditional knowledge practiced by the local people of Ethiopia. Multiple medicinal uses of HA inspired researchers to evaluate HA against different diseases through in vitro, in vivo, ex vivo, and clinical studies which strengthen the potential of HA for anti-parasitic, antibacterial, antifungal, antispasmodic, anticancer, anti-diabetes, antidiarrheal, wound healing, antioxidant, and anti-inflammatory activities (Figure 1).

### 6.1. Anti-Microbial Activity

Bacterial infectious diseases and drug resistance are serious threats to public health globally [24,25]. Plant-based antibacterial has emerged as a potential solution for bacterial drug resistance which demands the discovery of new plant-based antibacterial [26,27]. In traditional medicine, HA has been used against parasites as well as for bacterial infections. Different parts of the plant and various extract and Nano formulations prepared from HA have been studied in different studies to assess the antibacterial potential of HA against important bacterial species.

In an initial study, the antimicrobial activity of HA was studied with other medicinal plants to encounter the emerging problem of drug resistance reported in different bacteria [12]. In the study, the antibacterial and antifungal activities of extracts of HA were studied on both reference American-type cell culture and clinical isolates. Strong antibacterial activity extract of HA was observed against different important bacterial species in the agar diffusion method and minimum inhibitory concentration (MIC) calculation. Organic and aqueous extract of leaves and stem bark of the plant was studied and significant activities against *Staphylococcus aureus* (SA), Methicillin-resistant *Staphylococcus aureus* (MRSA), *Pseudomonas aeruginosa* (PA), *Microsporum gypseum* (MG), and *Trichophyton mentagrophytes* (TM) were observed in the study [12] (Table 2).

In the research, the antibacterial activity of extract flowers was studied. The researcher standardized the maceration method to minimize the possibility of thermal decomposition of thermo-labile components from the flower extract. Maceration in solvent for 72 h with intermittent shaking followed by filtering through cotton and filter paper was conducted. After other procedures, prepared extracts in different solvents such as methanol, ethanol, n-hexane, and petroleum ether were studied for yield and presence of phytochemicals. Finally, the methanol extract was the best yield and presence of different types of phytochemicals in the study compared with other solvents. The developed extraction method and the presence of phytochemicals have been used and correlated in further studies to study pharmacological properties such as anti-microbial anti-diabetes and wound healing activities [5,6,16]. Antibacterial activities of all extracts prepared in the study were studied through the agar well diffusion method against *Staphylococcus aureus* and *Salmonella typhi*. The methanolic extract was found to have the highest antibacterial activities against both bacterial species, i.e., *Staphylococcus aureus* and *Salmonella typhi* [28].

Later, in a study to explore optimal activity, the different parts of the plant, such as leaves, roots, and bark, were studied against SA and *Escherichia coli* (EC). The selected parts of the plant were used to extract the oil through different solvents (methanol, hexane, and ethyl acetate), and antibacterial activity was studied through the antibiotic diffusion method. The root extract had higher antibacterial activity compared with other parts (leaves and bark) of the plants [29]. Similarly, among extracts, the higher antibacterial activity was observed in methanol extract compared with other solvents (hexane and ethyl acetate) of the plants (Table 2).

Considering the effective antibacterial activities of HA and Cu nanoparticles (CuNPs) [36], the researcher synthesized CuNPs using HA leaf extract through green synthesis for enhanced antibacterial activity [33]. After the characterization, the synthesized CuNPs were used against selected gram-positive (SA and *Bacillus subtilis* (BS)) and negative (EC and PA) bacterial species. In the agar diffusion method, strong antibacterial activity of CuNPs was observed in all four studied pathogenic bacterial species, which was higher in the gram-positive species [33]. The capping and stabilization through the bioactive compounds present in the extract may be the important reason for the effective antibacterial activity of CuNPs.

The emerging role of nanomaterials in biological activity, such as antibacterial and antioxidant, inspired the researcher to synthesize the silver nanoparticles (AgNPs) from the leaves extract of HA. Bio-reduction of silver nitrate through extract was carried out for the green synthesis of AgNPs. The antibacterial properties of these synthesized AgNPs were studied through the agar well diffusion method against clinically isolated pathogenic bacterial species [30] (gram-negative bacteria (*Klebsiella pneumoniae* (KP)and *Salmonella typhimurium* (ST)) and gram-positive bacteria (*Streptococcus pneumonia* (SP)). Significant antibacterial activities were observed against all pathogenic bacteria, which were better than the extract alone and were highest in the case of ST (Table 2).

Similarly, considering the antibacterial activity of the extract of leaves of HA, the antibacterial activity of nanoparticles synthesized from the extract of leaves was also studied along with photochemical properties [31]. Synthesized zinc oxide nanoparticles from the aqueous extract of leaves were studied for antibacterial activity against SA, *Staphylococcus epidermidis* (SE), EC, and KP through the disc diffusion method. The antibacterial activities of NPs were observed against all bacteria, which was highest in SE.

The strong antibacterial activities of biosynthesized nanoparticles and nanocomposites (NCs) against reference, as well as drug-resistance bacteria, have been reported in the literature [37]. Thus, recently, researchers have studied the antibacterial properties of nanoparticles (NPs) and nanocomposites (NCs) prepared from the leaves of HA. Researchers synthesized the Ag and ZnO NPs, and Ag/bentonite, ZnO/bentonite, and Ag/ZnO/bentonite nanocomposites from extract of leaves for antibacterial activity against SA and EC. The antioxidant activity of Ag/ZnO/bentonite nanocomposite was found to be highest among different NPs and NCs used in the study in both minimum inhibitory concentration (MIC) and minimum bactericidal concentration (MBC) methods. The MIC and MBC were also calculated for Ag/ZnO/bentonite nanocomposite and found to be 78.125 and 156.25 μg/mL for SA [32]. Similarly, researchers synthesized magnesium oxide nanoparticles from the flower extract for antibacterial activity against gram-positive EC and gram-negative SA bacterial species. Significant antibacterial activity was observed in both strains, which was higher in the case of SA [34] (Table 2).

Like antibacterial, increased occurrences of resistance to conventional antifungal interventions make fungal diseases a public health concern worldwide. Plant-based antifungals have emerged as potential candidates for the new class of antifungal agents [38].

In a study, the medicinal plants reported to have antimicrobial activity were studied against the plant pathogen fungal species *Colletotrichum kahawae* [35]. Leaves Extract of HA was selected in the study based on its reported antimicrobial properties. Aqueous and ethanol extracts were studied against the selected pathogenic fungal species through an in vitro assay of radial growth in the petri dish. Moderate antifungal activity (60% inhibition) was observed in the case of aqueous extract, which was higher as compared to ethanol extract (Table 2) [35].

### 6.2. Anti-Parasite Activity of HA

Anti-parasite activity is an important medicinal property of HA, which has been utilized in traditional medicine for humans and different domestic animals such as goats and donkeys [35,39].

Helminthiasis is a disease caused by different macroscopic parasitic worms in animals and humans. It was estimated that around 2 billion people are infected with helminths globally. The parasite disease can also increase the complications and mortality along with other diseases [40,41]. The increasing reports of drug resistance in these parasitic worms ignite the development of natural therapeutics, which are considered to be potential solutions for the emerging drug resistance. 

#### 6.2.1. In Vitro Anti-Parasite Activity of HA

Essential oil from the plants has been reported to have several pharmacological activities, including trypanocidal activity. In a study, three medicinal plants, including HA, were selected for trypanocidal activities. The oil from the flowers was extracted through hydro distillation, and toxicity on *Trypanosoma brucei brucei* (TBB)cells was studied in 96 well plate assay. The essential oil of the flower was found to have trypanocidal activity (IC_50_ = 42.30 μg/mL) in the assay [18].

Later, the extract of female flowers of HA and its fraction according to polarity (n-heptane, ethyl acetate, and methanol) was studied for anthelmintic activity against four parasitic worms species, *Schistosoma mansoni* (blood fluke), *Echinostoma caproni* (intestinal fluke), *Clonorchis sinensis* and *Fasciola hepatica* (liver flukes). The n-heptane fraction was able to kill the worms in 3, 5, 17, and 1 h in the case of *Schistosoma mansoni*, *Clonorchis sinensis*, *Fasciola hepatica*, and *Echinostoma caproni*, respectively. The study revealed that the nonpolar fraction was more effective against the parasitic worms (Table 2). Further, researchers also developed screening assays for anthelmintic activities in non-pathogenic *Caenorhabditis elegans*, and the results were correlated with the observation found against the adult parasitic trematodes [22].

Recently, the anti-parasite activity of HA was studied to discover the phytochemicals of the plant responsible for its anti-parasite activity. The two types of fractions (ethanol crude extract and ethyl acetate fraction) of extract were used for the anti-trypanosoma activity by inhibiting TBB cells at two concentrations (30 and 60 μg/mL). The 100% inhibition of TBB cells at both low and high doses of ethyl acetate fraction was observed in the experiments; thus, this fraction was selected for further studies to discover the phytochemical present in this fraction for anti-parasite activity [21]. The acetylcholinesterase inhibiting activity of different fractions (n-hexane, dichloromethane, ethyl acetate, and water) of HA was also studied as acetylcholinesterase is considered as the important drug target for anti-parasite activity. The maximum acetylcholinesterase inhibition was observed for ethyl acetate fraction, like anti-trypanosoma activity. Further, to discover the phytochemicals of the ethyl acetate fraction for their anti-parasite activity, screening of phytochemicals found to be present in the ethyl acetate fraction was carried out against lactate dehydrogenases, acetylcholinesterase, and glutathione reductase which are the important targets enzymes for anti-parasite activity. The affinity interactions of selected target enzymes with phytochemicals identified in the fraction of HA were analyzed through the UF-LC–MS method. The corilagin has shown the highest potential against acetylcholinesterase as its binding degree was highest among other phytochemicals identified in the extract, followed by quercetin. Similarly, methyl brevifolin carboxylate has shown the highest potential against other target enzymes (lactate dehydrogenases and glutathione) as its binding degree was highest among other phytochemicals [21].

Additionally, a molecular docking study was conducted to analyze the binding affinity and mode of binding for identified phytochemicals against all three selected drug target enzymes, i.e., lactate dehydrogenases, acetylcholinesterase, and glutathione [21]. 

#### 6.2.2. In Vivo Anti-Parasite Activity of HA

Later, inspired by the traditional use of HA for anthelmintic activities in animals and the presence of anthelmintic substances in HA, the anthelmintic activity of HA was studied in the Alpine goats [42]. Three doses (20, 40, and 60 mg) of dried material of HA were orally given to the goats, and eggs in feces were studied to examine the anthelmintic activity. In all doses, the cestode egg counts were reduced in the study. The reduction in cestode egg counts was higher in 20 and 40 mg doses as compared to the highest dose (60 mg), suggesting further study may be required to optimize the treatment dose and identify the possible mechanism behind the anthelmintic activity of HA [42]. 

#### 6.2.3. Anti-Parasite Activity on Human Subjects

In a study to screen medicinal plants for anti-parasite activity, the toxicity and the dosage of traditionally used herbal medicine against the parasitic worm *Taenia saginata* were studied with 33 known anti-parasite plants. HA is included in the study as the use of flowers of HA is among the popular herbal medicines against Helminthiasis in Ethiopian peoples [1]. In the toxicity study, the adult albino mice were used for IP dosage, and LD_50_ was calculated for each plant [14]. The oral dose of powdered flowers with honey was administered to the volunteers in groups of six per person. The median effective single dose and worm expulsion time were calculated and found to be 12.5 ± 2.2 mg/kg and 11.3 ± 1.4 h in the case of HA. HA was found to be 3rd and 4th best in the cases of median effective single dose and worm expulsion time, respectively (Table 3). The study concluded that further research might be conducted to develop top-ranking plants such as HA as therapeutic against parasitic worms. 

### 6.3. Antidiarrheal Activity

Studies have highlighted the potential of plants as a source of antidiarrheal therapeutics [46,47]. HA is the most commonly used traditional medicine for diarrhea in Ethiopia. Thus, the study is conducted to validate the antidiarrheal potential and safety of HA to develop this plant as therapeutics against diarrhea. The methanol crude extract of leaves and the fraction of extract in aqueous, ethyl acetate, and chloroform solvents were studied for antidiarrheal activity on castor oil-induced animal diarrhea models. The Swiss albino mice were used in the animal studies. Different diarrhea-related parameters, such as in vivo antidiarrheal index, inhibition of defecation, gastrointestinal motility, and enteropooling, were studied in the castor oil-induced diarrhea model. Antidiarrheal activity on different studied parameters was improved in a dose-dependent manner in all extract fractions of HA leaves. The aqueous fraction of extract was found to have higher activity values in inhibition of defecation and gastrointestinal motility as compared to crude, ethyl acetate, and chloroform extracts. Similarly, higher antidiarrheal index values were observed in the crude extract as compared to the fraction of extract in aqueous, ethyl acetate, and chloroform solvents, while the ethyl acetate fraction had the higher activity in inhibition of castor oil-induced intestinal enteropooling as compared to crude, aqueous and chloroform extracts [13,43]. The study not only supports antidiarrheal usage of HA but provides the path to the development of HA as an antidiarrheal candidate in future research.

### 6.4. The Anti-Spasmodic Activity of HA

Anti-spasmodic drugs are used to relieve spasms and cramps in the stomach and intestine, which may be associated with different diseases of the digestive system. In an initial study, the anti-spasmodic effect of female flowers of HA was studied on the guinea pig ileum model. The water extract of the female flowers was found to antagonize the effect of three spasmogens (acetylcholine, histamine, and barium chloride) used in the study in separate experiments. The dose cumulative graph of the activity indicated the noncompetitive inhibition of spasmogen activity through the extract. The study suggests the extract of female flowers can reduce the contraction of guinea pig ileum induced by the selected spamogens. Further study to analyze individual constituents of the extract for antispasmodic activity may be conducted in the near future [44].

### 6.5. Anti-Cancer Effects of HA

Like diabetes, the escalation in the incidence of cancer is also growing globally. Due to relatively less toxicity and abundant availability, natural therapeutics are considered important potential anticancer agents [48,49]. Literature also supports the anticancer activity of plant products, and the anticancer activity of HA was also reported in traditional medicine of HA along with other medicinal plants.

#### 6.5.1. In Vitro Anti-Cancer Effects of HA

In an initial study, the anticancer activity of kosins isolated from the female flowers of HA was studied through an in vitro tumor cell line and in vivo tumor mice model. Murine adenocarcinoma of the colon 15A (MAC 15A) cell line was used to study the in vitro toxicity of kosins against cancerous cells. The results showed that all three kosins (α-kosin, kosotoxin, and protokosin) used in the study were anti-proliferative against MAC 15A cells with IC_50_ ranges between 3 to 5 µM [45].

The anti-cancer activity of essential oil from the female flowers of HA was studied through anti-leukemia activity observed against the HL-60 cell line after studying its phytochemicals through gas and liquid chromatography-mass spectrometry (GLCMS). The oil from the flowers was extracted through the hydro distillation process and found to inhibit the HL-60 cell with IC_50_ = 50.07 μg/mL [18]. This study can be considered as primarily in nature, which suggests the anticancer potential of HA.

Later, owing to the antioxidant activity of HA and reported use of roots along with other medicinal plants for the treatment of cancer in traditional medicine. The anticancer activity of roots of HA was studied through 3-(4,5-dimethylthiazol-2-yl)-2,5-diphenyltetrazolium bromide (MTT) assay on three different cell lines, HepG2, HT-29, and SGC-7901 which corresponds to liver cancer, colon cancer, and gastric cancer [2]. Like previous studies on female flowers, the researcher standardized the maceration method for the preparation of extract from the roots, possibly to minimize the thermal decomposition of thermo-labile components [28]. The maceration with 95% ethanol (3 times, 2 d/time) at room temperature, followed by the extraction with different solvents, such as n-hexane, dichloromethane, and ethyl acetate, was used in sequence to obtain corresponding extracts. Finally, the ethyl acetate extract was found to have the highest TPC and TFC compared with other solvents and selected for further studies. Similarly, the anti-proliferative activity observed in the study was higher in ethyl acetate and hexane fractions in all studied cell lines [2].

#### 6.5.2. In Vivo Anti-Cancer Study of HA

Researchers have analyzed the anticancer effect of kosins isolated from the female flowers of HA through an in vivo tumor mice model after positive screening results from in vitro cell line study [45]. NMRI mice were used for anti-tumor activity after transplantation of well-differentiated murine adenocarcinoma of the colon (MAC) tumors. The single dose of 50 mg/kg and split doses of 12.5 mg/kg (for 4 days) of kosins (α-kosin, kosotoxin, and protokosin) isolated from HA given to mice through intraperitoneal administration. No antitumor activity was observed in the groups that received a single dose of kosins, but in split dose groups, moderate antitumor activity (increased in the survival time) was observed in the groups that received kosotoxin and protokosin. The study has shown the potential antitumor activity of two kosins (kosotoxin and protokosin), which must be investigated in further experiments for their development as anticancer candidates [45].

### 6.6. Antioxidant Activity of HA

The antioxidant property is considered one of the important properties that can contribute to multiple pharmacological properties, including anticancer activities [50]. The presence of important phytochemicals known for antioxidant activity, such as phenols, saponins, flavonoids, anthraquinones, terpenoids, alkaloids, steroids, glycosides, and tannins, may explain the antioxidant properties of HA. The reported anticancer use of roots of HA along with other medicinal plants in Kofele by local doctors also indicated the anticancer potential of HA [1].

The antioxidant activity of roots of HA was studied through different methods, including 2,2-diphenyl-1-picryl-hydrazyl free radical (DPPH) scavenging, ABTS^+^ radical cation scavenging activity, and Ferric-Ion reducing antioxidant power (FRAP) assays [2]. The methanol extract of HA roots and its fraction in water, n-hexane, dichloromethane, and ethyl acetate were analyzed. The antioxidant activity was highest in the case of ethyl acetate fraction in DPPH and ABTS assays, which was better than the positive control used in the study, while the FRAP value was higher in the case of ethanol extract followed by the ethyl acetate fraction. Further, the chemical constituent of ethyl acetate fraction was studied due to the higher antioxidant activity of this fraction in both DPPH and ABTS fractions. The compounds important in antioxidant activity were isolated from the active fraction of the extract and studied for antioxidant activity through previous methods (DPPH, ABTS, and FRAP assays). Among isolated compounds, caffeic acid had the strongest antioxidant activity, which was better than the positive control in the DPPH assay. Similarly, the quercetin had the strongest antioxidant activity among other isolated compounds in ABTS and FRAP assays.

The emerging role of nanomaterials in biological activity, such as antioxidants, inspired the researcher to synthesize the silver nanoparticles (AgNPs) from the leaves extract of HA (LEHA). Bio-reduction of silver nitrate through LEHA was carried out for the green synthesis of AgNPs. The antioxidant properties of these synthesized AgNPs were studied through 2,2-diphenyl-1-picryl-hydrazyl (DPPH) radical scavenging activity. In the DPPH assay, the dose-dependent antioxidant activity was observed, which was highest at 320 μg/mL concentration of AgNPs [30].

The extract preparation method for the leaves was standardized according to the previously reported extraction method for flowers. The leaves were powdered with an electric mill after drying at room temperature, which was macerated distinctly in 80% methanol for 3 days. After other procedures, the methanol extract was also fractioned with other solutions, including Ethyl acetate, chloroform, and aqueous fractions. The percentage yield and actual yield were highest in aqueous and methanol extracts, respectively [5]. The methanol extract from leaves and the fraction of extract in water, ethyl acetate, and chloroform were studied using DPPH inhibition assay. The concentration-dependent antioxidant activities of extract and fraction were observed, which were highest in the case of crude extract [5].

### 6.7. Antidiabetic Activity of HA

The prevalence of diabetes is escalating worldwide. In 2021, more than 500 million people were estimated to have diabetes, which poses a challenge to public health [51]. Herbal medicine is an important resource for potential therapeutic against diabetes that is used by the local population [52,53]. Different parts of HA have been used in traditional medicine for the management of various diseases, including diabetes, in different countries in the African continent. In a hospital-based cross-sectional study in Ethiopia, HA was found to be one of the most used herbal medicines among diabetic persons in a teaching hospital [52]. In later studies, the anti-diabetes potential of different parts of HA has been analyzed through in vitro and in vivo experiments.

#### 6.7.1. In Vitro Anti-Diabetes Studies on HA

Motivated by the use of HA leaves in Ethiopian folk medicine, researchers studied the anti-diabetes activity of HA leaf extract through in vitro inhibition of α-amylase and α-glucosidase enzymes. The methanol extract and the fraction of extract in water, ethyl acetate, and chloroform were studied using 3,5-dinitrosalicylic acid (DNSA), p-nitro-phenyl-a-D glucopyranoside (p-NPG) assays. The highest inhibitory activities for α-amylase and α-glucosidase enzymes were observed in the case of crude extract and water fraction, respectively. They revealed the antidiabetic potential of HA leaves through the inhibition of α-amylase and α-glucosidase enzymes [5]. The researchers studied the antidiabetic activity of flowers of HA in both in vitro and in vivo studies, along with antioxidant activity. In vitro α-amylase inhibition assay was conducted through the DNSA method for crude extract and its fraction in different solvents (water, ethyl acetate, and chloroform) along with acarbose as the positive control [16]. The inhibitory activities were observed in all extracts and fractions in a dose-dependent manner, which was higher in ethyl acetate fraction as compared to crude extract and water and chloroform fractions (Table 2).

#### 6.7.2. In Vivo Anti-Diabetes Studies on HA

The methanol extract of leaves of HA was used to study anti-diabetes activity in animal studies after the phytochemical estimation analysis [23]. Three doses (100, 200, and 400 mg/kg) extract of leaves were selected from the acute toxicity study and administered to the three groups of animals along with normal, negative, and positive control groups. Blood glucose level and body weight were examined weekly in a 14-day study. A significant reduction in blood glucose levels was observed in all doses of leaf extract as compared to the diabetic control group and baseline at both the 7th and 14th days of the experiment. Similarly, a significant improvement in body weight was observed in the high-dose (400 mg/kg) leaf extract group as compared to the diabetic control group on the seventh day of the experiment. On the 14th day of the experiment, significant improvement in body weight was observed in the middle (200 mg/kg) and high-dose (400 mg/kg) of leaf extract as compared to the diabetic control group and baseline (within group). The effect of extract from HA was also studied on serum plasma level on the 15th day of the experiment. A significant improvement (reduction) in serum total cholesterol in high and middle doses of leaf extract was observed as compared to the diabetic group. Meanwhile, a reduction in serum triglyceride, very low-density lipoprotein, and low-density lipoprotein cholesterol was observed in all doses of extract. Improvement (increase) in high-density lipoprotein cholesterol level was also observed in all doses of extract as compared to the diabetic group.

In the case of flowers, the animal studies were also conducted according to results obtained from the in vitro studies [16]. In the normoglycemic mice in vivo model, the hypoglycemic activity of crude extract of flower was studied at 0 (baseline), 1, 2, 4, and 6 h for 100, 200, and 400 mg/kg concentrations along with positive and negative controls. A significant reduction in blood glucose level was observed in 6 h as compared to negative control at 200 and 400 mg/kg concentrations. Similarly, in the oral glucose tolerance test on normoglycemic mice, a significant reduction in blood glucose level was observed after 120 min as compared to negative control at 200 and 400 mg/kg concentrations. Further, crude methanol extract and its fraction in different solvents (water, ethyl acetate, and chloroform) were also studied on the STZ-induced diabetic mice model with a single dose for anti-hyperglycemic activity. In the single-dose study, the reduction in the blood glucose levels was higher in 400 mg/kg doses in crude extract and fractions, but a significant reduction was observed in crude extract and ethyl acetate fraction in comparison with negative control at the eighth hour. Thus, the repetitive doses of crude extract and ethyl acetate fraction were also studied for anti-hyperglycemic activity, effect on body weight, lipid level in STZ-induced diabetic mice model, and weekly blood glucose level. In both crude extract (at 200 and 400 mg/kg) and ethyl acetate (at 200 and 400 mg/kg) fraction administered groups, the blood glucose levels were significantly reduced as compared to the control group on the 7th and 14th days. Within a group study, the significant reduction of blood glucose levels was also found to be significant in crude extract (400 mg/kg) and ethyl acetate (400 mg/kg) fraction as compared to baseline level. The effect of treatment on body weight in the streptozotocin-administered mice was also studied. In comparison with diabetic control, a significant increase in the body weight of mice was observed on the 7th day in 200 and 400 mg/kg doses of crude extract and 400 mg/kg dose of ethyl acetate fraction groups. A significant increase in the body weight of mice was observed in all doses of crude and ethyl acetate fraction administered groups on the 14th day. Within groups, a significant increase in body weight compared to baseline was observed in the 400 mg/kg crude extract administered group and 200 mg/kg ethyl acetate administered groups. Similarly, serum lipid profile was also studied in these groups, and serum levels of TC, TG, LDL, and VLDL were found to be significantly reduced in all doses of crude extract and 400 mg/kg of ethyl acetate fraction administered groups as compared to the diabetic control group. The levels of HDL-c were found to be significantly increased in all doses of crude extract and 200 and 400 mg/kg of ethyl acetated fractions [16] (Table 3).

### 6.8. Wound Healing Effects of HA

Traditionally, the flowers and leaves of HA have been used for wound healing in local populations, but the scientific study for wound-healing activity was not reported. The wound-healing potential of a series of plants has been verified in experimental studies [54,55]. Thus, the study was conducted to analyze the wound-healing activity of HA in different animal wound-healing models. The methanol extract of flowers and its fraction in chloroform, ethyl acetate, and water was prepared as ointments (5 and 10% *w*/*w*) to study wound-healing activity. Dermal toxicity was also studied in mice animal models before the wound-healing experiments. Swiss albino mice were used for incision and excision wound models to evaluate the wound-healing activity of crude extract and fractions. The ointment prepared with crude extract significantly increased wound contraction from day 4th to 14th at both doses (5 and 10% *w*/*w*). The wound closure was 100% in positive control and 10% *w*/*w* dose of crude extract on the 14th day [6]. Meanwhile, 100% wound closure was observed on the 16th day at 5% *w*/*w* ointment application dose of crude extract. Significantly, improved wound contraction was also observed in the treatment with different fractions. Among the fractions, the highest wound contraction was observed in the case of ethyl acetate fraction at 10% *w*/*w* dose. In wound healing, the epithelization period was also found to be shortened in 5 and 10% crude extract ointment application groups compared with negative control. The high-dose crude extract (10% *w*/*w*) had the shortest epithelization period, surpassing the positive control. Similarly, a significant reduction of the epithelization period was also observed in the treatment with different fractions. Among the fractions, the shortest epithelization period was observed in the case of ethyl acetate fraction at 10% *w*/*w* dose.

In the incision wound model, the tensile strength was significantly increased in (both 5 and 10%) crude extract ointments and positive control groups compared with negative control group. 10% *w*/*w* dose crude extract treatment group and positive control had comparable tensile strength in the experiment, which suggests a strong wound-healing potential of the flower and supports the traditional wound-healing usage of HA [6].

### 6.9. Anti-Inflammatory Activity of HA

Like antioxidant activity, anti-inflammatory activity can contribute positively to different diseases, including cancer. Along with wound healing activity, the anti-inflammatory activity of HA was studied through the carrageenan-induced hind paw edema model in mice [6]. Flowers of HA were used for the preparation of methanol extract to study the anti-inflammatory activity. Three oral doses of 100, 200, and 400 mg/kg were selected with positive control (indomethacin) for the study, and a reduction in paw edema was observed in all doses of HA and positive control in the study [6]. Secondary metabolites such as flavonoids were speculated to be responsible for the inhibition of paw edema, which was in a dose-dependent manner.

## 7. Results Gaps and Future Direction

HA is a multipurpose tree plant with several potential medicinal properties which has been used by the local population. These major medicinal properties of HA are also supported through experiments that mark HA highly important, demanding more research and development exertions to establish the medicinal properties for possible health-promoting usages. However, the diverse medicinal properties of the plant have different challenges.

The anti-parasite activity of HA has been utilized for both humans and animals in traditional medicine. Various studies found the anti-parasite activity of HA against several parasites, but still, limited clinical studies have been conducted. An initial study on humans infected with *Taenia saginata* validated its activity; however, more clinical studies will be required to optimize the anti-parasite activity of HA against the different parasites. Limited efforts have been made to identify the drug target/mechanism of action of the extract from HA or its constituent. Further research to decipher the mechanism or drug target is required to achieve optimal anti-parasite activity against different pathogenic parasites. This may help to develop HA as a candidate therapeutic, followed by clinical experiments after safety studies on animals. 

Similarly, an experimental study validated the wound-healing activity that HA has been used in the local population. Still, more studies are suggested to optimize the wound-healing activity of HA through different animal models before clinical trials to develop it.

Antibacterial activity of HA through the different types of extract and formulation, including Nano-particles against numerous pathogenic bacteria, has been observed in several studies, which makes antibacterial activity an important activity to be developed as a therapeutic candidate. In contrast to antibacterial activity, the antifungal activity of HA is not well studied. The antifungal activity was only analyzed on one plant pathogen fungal species, which is considered to be moderately active [35]. Antifungal studies against other important fungal species (such as *Candida* or *Aspergillus* species) are suggested to be conducted to verify the antifungal potential of HA before considering it a potential antifungal candidate.

Antispasmodic activity is also one of the important activities HA based on traditional knowledge, but it was only validated in one ex vivo study. Hence, more studies may be carried out to establish the antispasmodic activity of HA for further consideration in clinical studies.

The anticancer activity of HA was studied through in vitro as well as in vivo models. Still, limited studies have been conducted to decipher the molecular mechanism of anticancer activity, which restricts the optimal and reliable use of the anticancer potential of HA. Further studies are suggested to decipher the molecular mechanism of HA intervention for anticancer activities. One prospective mechanism may be through anti-inflammatory pathways for anticancer activity, as HA was found to be anti-inflammatory in animal models, which also potentiates its anticancer activity.

Anti-inflammatory activity was observed through animal models, and like anticancer activity, no mechanistic study has been conducted to decipher the possible mechanism behind the anti-inflammatory activity. Knowledge of the action mechanism is highly required to establish this anti-inflammatory activity for optimal therapeutic development. 

Antidiarrheal activity of HA is which can be considered an important positive effect on the gastrointestinal system along with other activities. The studies have supported the traditional use of HA as antidiarrheal based on an animal model. It is also suggested to analyze the antidiarrheal effect of HA on other animal models. 

Like anti-inflammatory activity, antioxidant activity is also known to contribute to different pharmacological activities, including cancer. Antioxidant activity of HA was markedly observed in several studies through different methods, which made this activity a highly prospective activity of HA to work on [2,5,56,57]. The gap in the development of the antioxidant potential of HA is the limited antioxidant activity in in vivo and clinical studies, which may be considered in future studies to harness the antioxidant potential of HA as therapeutic.

HA was found to be the most used herbal intervention among local people suffering from diabetes [52], and experimental studies support that in the case of both leaves and flowers [16,23]. The studies also highlight the importance of phytochemicals of HA responsible for antidiabetes activity, as the difference in activity was observed in extract types against important target enzymes. Thus, studies to identify the phytochemical component responsible for the anti-diabetes effect are highly suggested to design optimal dosage for clinical studies for the development of HA as an herbal medication for diabetes. Overall, the reported studies are lacking in deciphering the molecular mechanism of action responsible for almost all reported pharmacological activities of HA. Therefore, studies to understand the molecular mechanism of action responsible for different pharmacological activities of HA are strongly suggested. It would help optimize the pharmacological activities of HA.

Studies also revealed the presence of diverse classes of phytochemicals such as alkaloids, polyphenols (tannins, flavonoids, and anthraquinones), and terpenoids (saponins and steroids) in different parts of HA. Mostly, the pharmacological activities of HA were studied inspired by traditional knowledge without considering the information on the presence of phytochemicals. It can be considered as a limitation of current studies to discover the full pharmacological potential of HA. The chemical characterization of the extract was also missing in some studies, which is one of the most important aspects of the pharmacological properties of the extract. Chemical characterization of extracts is highly suggested in future studies as it can help in optimizing the doses and pharmacological activities in further animal studies. The compounds identified in HA, such as rutin, quercetin, acacetin, gurjunene, curcumene, and brevifolin, are known for various other medicinal activities, especially neuroprotective and cardio-protective properties [58,59]. The presence of these phytochemicals indicates the potential for neuroprotective activity of HA [60], which is not studied in the literature. Thus, the neuroprotective and cardio-protective potential of HA is strongly suggested to be investigated in future studies. The reported antioxidant activity of HA also supports the possible neuroprotective activity of HA. Considering the available information, it can be concluded that HA has the huge potential to develop as a therapeutic against different diseases and conditions. The suggestion and direction provided in the current work may be helpful for the development of HA not only for known activities but also for proposed diseases and conditions in future studies.

## Figures and Tables

**Table 1:** Phytochemicals reported in HA.

Sr. No.	Compound Name/Molecular Formula	Amount (µg/g)	Compound Type	Type of Extract/Fraction	Part of Plant	References
1	p-Hydroxybenzoic acid/C_7_H_6_O_3_	8.92	Phenolic acids	Ethyl acetate	Male Flower	[17]
		4.27	Phenolic acids	Ethyl acetate	Female flower	[17]
2	Protocatechuic acid/C_7_H_6_O_4_	4.51	Phenolic acids	Ethyl acetate	Male Flower	[17]
		4.86	Phenolic acids	Ethyl acetate	Female flower	[17]
3	Vanillic acid/C_8_H_8_O_4_	9.93	Phenolic acids	Ethyl acetate	Male Flower	[17]
		5.39	Phenolic acids	Ethyl acetate	Female flower	[17]
4	α-Kosin/C_25_H_32_O_8_	220	Phenol derivative	Diethyl ether	Male Flower	[17]
		240	Phenol derivative	Diethyl ether	Female flower	[17]
5	Kosotoxin/C_25_H_32_O_8_	1080	Phenol derivative	Diethyl ether	Male Flower	[17]
		960	Phenol derivative	Diethyl ether	Female flower	[17]
6	Protokosin/C_37_H_46_O_12_	760	Phenol derivative	Diethyl ether	Male Flower	[17]
		720	Phenol derivative	Diethyl ether	Female flower	[17]
7	Corilagin/C_27_H_22_O_18_	NP	Tannin	Ethyl acetate fraction	Root	[21]
8	Brevifolin carboxylic acid/C_13_H_8_O_8_	NP	Phenol derivative	Ethyl acetate fraction	Root	[21]
9	Brevifolin/C_10_H_12_O_4_	NP	Phenolic compound	Ethyl acetate fraction	Root	[21]
10	Methyl brevifolin carboxylate/C_14_H_10_O_8_	NP	Phenol derivative	Ethyl acetate fraction	Root	[21]
11	Quercetin/C_15_H_10_O_7_	NP	Flavonoid	Ethyl acetate fraction	Root	[21]
12	Methyl ellagic acid/C_15_H_8_O_8_	NP	Polyphenol	Ethyl acetate fraction	Root	[21]
13	Yomogi alcohol/C_10_H_18_O	1.11 ^#^	Terpenoid	Essential oil	Female flower	[18]
14	Camphenone/C_10_H_14_O	0.32 ^#^	Monoterpenoid ketone	Essential oil	Female flower	[18]
15	L-Camphor/C_10_H_16_O	2.16 ^#^	Monoterpenoid ketone	Essential oil	Female flower	[18]
16	Limonene oxide/C_10_H_16_O	0.68 ^#^	Monoterpenoid epoxide	Essential oil	Female flower	[18]
17	Cis-verbenol/C_10_H_16_O	3.27 ^#^	Monoterpenoid alcohol	Essential oil	Female flower	[18]
18	Trans-verbenone/C_10_H_14_O	0.38 ^#^	Monoterpenoid ketone	Essential oil	Female flower	[18]
19	α-Phellandren-8-ol/C_10_H_16_O	3.68 ^#^	Monoterpenoid alcohol	Essential oil	Female flower	[18]
20	Gurjunene/C_15_H_24_	1.15 ^#^	Sesquiterpene	Essential oil	Female flower	[18]
21	Curcumene/C_15_H_22_	0.58 ^#^	Sesquiterpene	Essential oil	Female flower	[18]
22	α-Selinene/C_15_H_24_	0.45 ^#^	Sesquiterpene	Essential oil	Female flower	[18]
23	Valeranone/C_15_H_26_O	10.58 ^#^	Sesquiterpenoid ketone	Essential oil	Female flower	[18]
24	Palustrol/C_15_H_26_O	5.70 ^#^	Sesquiterpenoid alcohol	Essential oil	Female flower	[18]
25	Ledol/C_15_H_26_O	58.57 ^#^	Sesquiterpenoid alcohol	Essential oil	Female flower	[18]
26	Hexadecen-1-ol/C_16_H_32_O	2.59 ^#^	Fatty alcohol (Lipid)	Essential oil	Female flower	[18]
27	Trans-9-E-15-Heptadecenal/C_17_H_32_O	4.45 ^#^	Fatty aldehyde	Essential oil	Female flower	[18]
28	Tetracosane/C_24_H_50_	2.07 ^#^	Alkane	Essential oil	Female flower	[18]
29	Diallyl methyl carbinol/C_8_H_14_O	0.99 ^#^	Terpenoid alcohol	Essential oil	Female flower	[18]
30	3-Pinanylamine/C_10_H_19_N	0.46 ^#^	Terpene amine	Essential oil	Female flower	[18]
31	2-Furanmethanol/C_5_H_6_O_2_	0.44 ^#^	Alcohol	Essential oil	Female flower	[18]
32	Dihydroquercetin/C_15_H_12_O_7_	NP	Flavonoid	Ethanol extract	Root	[2]
33	Acacetin/C_16_H_12_O_5_	NP	Flavonoid	Ethanol extract	Root	[2]
34	Isoquercitin/C_21_H_20_O_12_	NP	Flavonoid glycocide	Ethanol extract	Root	[2]
35	Dehydrodicatechin A/C_30_H_24_O_12_	NP	Flavonoid	Ethanol extract	Root	[2]
36	Trans-ferulic acid/C_10_H_10_O_4_	NP	Phenol	Ethanol extract	Root	[2]
37	Caffeic acid/C_9_H_8_O_4_	NP	Polyphenol	Ethanol extract	Root	[2]
38	3,4-Dihydroxybenzoic acid/C_7_H_6_O_4_	NP	Polyphenol	Ethanol extract	Root	[2]
39	2-Methoxyterephthalic/C_9_H_8_O_5_	NP	Aromatic acid	Ethanol extract	Root	[2]
40	Quercetin 3-O-β-glucuronide/C_21_H_18_O_13_	NP	Flavonoid glycoside	Methanol extract	Female flower	[22]
41	Quercetin 3-O-β-glucoside/C_21_H_20_O_12_	NP	Flavonoid glycoside	Methanol extract	Female flower	[22]
42	Rutin/C_27_H_30_O_16_	NP	Polyphenolflavonoid glycoside	Methanol extract	Female flower	[22]
43	Quercetin glycuronide/C_21_H_18_O_13_	NP	Flavonol glucuronide	Methanol extract	Female flower	[22]
44	Ellagic acid/C_14_H_6_O_8_	NP	Polyphenol	Methanol extract	Female flower	[22]

^#^: % of each component among essential oil; NP: not provided.

**Table 2:** Pharmacological activities of HA studies in vitro studies.

Sr. No.	Activity	Plant Part/Extract Type	Method	Result	Ref.
1	Antibacterial	Organic (hexane, DCM and methanol) and aqueous extract of leaves	Mean inhibition zones (MIZ) caculation through agar diffusion method against different bacteria	MIZ in hexane: 6.67 ± 0.67, 19.33 ± 1.33, 13.00 ± 1.00, and 6.83 ± 0.17 mm against SA, MRSA, PA, and TM, respectively. MIZ in DCM: 20.00 ± 1.15, 19.50 ± 1.50, 15.5 ± 1.50, and 7.17 ± 0.44 mm against SA, MRSA, PA, and TM, respectively. MIZ in methanol: 7.75 ± 0.25, 7.63 ± 0.24, and 12.00 ± 0.82 mm against SA, MRSA, and PA, respectively.	[12]
			Minimum inhibitory concentration (MIC) against different bacteria	MIC in hexane: 0.195, 0.195, and 3.125 against SA, MRSA, and PA, respectively. MIC in DCM: 0.195, 0.391, and 0.195 mg/mL against SA, MRSA, and PA, respectively. MIC in Methanol: 12.5 mg/mL against PA;	
		Organic (hexane and methonol) and aqueous extract of stem bark	MIC against different bacteria	MIC in hexane: 6.25 and 6.25 mg/mL against SA and MRSA, respectively. MIC in DCM: 100 and 100 mg/mL against SA and MRSA, respectively. MIC in methanol: 25 and 50 mg/mL against SA and MRSA, respectively.	
			MIZ against different bacteria	MIZ in hexane: 9.00 ± 1.00, 9.50 ± 0.96, and 7.00 ± 0.33 mm against SA, MRSA, and TM, respectively. MIZ in DCM: 8.67 ± 0.33, and 9.33 ± 0.67 mm against SA and MRSA, respectively. MIZ in methanol: 11.00 ± 0.41, 10.75 ± 0.25, 12.25 ± 1.75, and 7.33 ± 0.33 mm SA, MRSA, PA, and MG, respectively. Water: 11.33 ± 0.67 mm against PA;	
		Extract of female flowers in methanol, ethanol, n-hexane, and petroleum ether	Agar well diffusion method.	SA: 21, 20, 15, and 14 mm for methanol, ethanol, n-hexane, and petroleum ether, respectively. ST: 15, 14, 14, and 12 mm for methanol, ethanol, n-hexane, and petroleum ether, respectively.	[28]
		Oil extracted from the root through methanol, ethyl acetate, and hexane.	Antibiotics diffusion method against SA and EC	Ethyl acetate: 8.87 and 8.75 mm in case of SA and EC, respectively. Hexane: 9.87 and 5.87 in case of SA and EC, respectively. Methanol: 24.38 ad 27.13 in case of SA and EC, respectively.	[29]
		Oil extracted from the leaves through methanol, ethyl acetate and hexane	Antibiotics diffusion method against SA and EC	Ethy acetate: 8.38 and 9 mm in case of SA and EC, respectively. Hexane: 20 and 6.5 in case of SA and EC, respectively. Methanol: 12 ad 12.5 in case of SA and EC, respectively.	
		Oil extracted from the root bark through methanol, ethyl acetate and hexane	Antibiotics diffusion method against SA and EC.	Ethyl acetate: 8.75 and 6.75 mm in case of SA and EC, respectively. Hexane: 7.5 and 4.88 in case of SA and EC, respectively. Methanol: 15.37 ad 1.125 in case of SA and EC, respectively.	
		extract of leaves and Silver nanoparticles synthesized from the extract (50, 100, 150, and 200 μg/mL)	Agar well diffusion method.	AgNPs: 18.3, 14, and 8.6 in case of ST, KP, and SP, respectively. Leave extract: 13, 10, and 6.3 in case of ST, KP, and SP, respectively.	[30]
		Zinc oxide nanoparticles from the aqueous extract of leaves. (10, 20, and 30 mg/mL)	Disc diffusion method 2,3,5-triphenyltetrazolium chloride	19 ± 1.0, 18 ± 1.0 19.33 ± 0.58, and 21 ± 1.0 mm in case of EC, KP, SA, and SE, respectively.	[31]
		Ag NPs, Ag/bentonite NCs, Ag/ZnO/bentonite, ZnO NPs, and ZnO/bentonite nanocomposites prepared from the leaves extract	Disc diffusion method	Ag NPs: 11.6 ± 0.3 and 14.3 ± 0.3 mm in case of EC and SA, respectively. Ag/bentonite NCs: 14.3 ± 0.1 and 14.7 ± 0.3 mm in case of EC and SA, respectively. Ag/ZnO/bentonite:14.3 ± 0.3 and 17.3 ± 0.2 mm in case of EC and SA, respectively. ZnO NPs: 10.3 ± 0.3 and 11 ± 0.6 mm in case of EC and SA, respectively. ZnO/bentonite: 12.3 ± 0.3 and 12.3 ± 0.3 mm in case of EC and SA, respectively. Plant extract: 9.1 ± 0.3 and 10 ± 0.0 mm in case of EC and SA, respectively.	[32]
		Copper nanoparticles synthesized from the extract of leaves	Agar disc-diffusion method Ampicillin	12.7 ± 0.4, 14.7 ± 0.2, 14.2 ± 0.8, and 12.7 ± 1.1 mm in case of EC, SA, BS, and PA, respectively.	[33]
		Ag/ZnO/bentonite nanocomposite prepared from the leaves extract	MIC and MBC were calculated through Broth dilution methods	MIC: 156.25 and 78.125 μg/mL for EC and SA, respectively. MBC: 312.5 and 156.25 for EC and SA, respectively.	[32]
		MgO nanoparticles synthesized from the aqueous extract of the flowers	Agar-well-diffusion method (0.8 mg/mL) chloramphenicol (30 μg)	15 ± 0 and 27 ± 0.28 mm for EC and SA, respectively.	[34]
2	Antioxidant	Methanol extract of HA roots and its fraction in water, n-hexane, dichloromethane, and ethyl acetate	DPPH	IC_50_ values 99.700 ± 0.013 g/mL (Ethyl acetate) and 98.680 ± 0.010 (Trolox)	[2]
			ABTS	31.200 ± 0.001 g/mL (Ethyl acetate) and 64.760 ± 0.003 g/mL (Trolox)	
			FRAP	3.478 mg Fe^2+^/g (ethanol extract)	
		Silver nanoparticles synthesized from the extract of leaves and extract of leaves (10–320 μg/mL)	Percentage inhibition DPPH radical scavenging activity (ascorbic acid as control)	66% in AgNPs and 95.9% in control	[30]
		Methanol extract of leaves and solvent fractions (water, ethyl acetate, and chloroform) 15.6–500 μg/mL	percentage inhibition DPPH radical scavenging activity (ascorbic acid as control)	86.36% (IC_50_, 10.25 μg/mL) followed by water fraction 78.59% (IC_50_, 13.86 μg/mL), ethyl acetate fraction 71.58% (IC_50_, 16.34 μg/mL), andchloroform fraction 63.65% (IC_50_, 18.83 μg/mL).	[5]
3	Antifungal	Aqueous and ethanol extracts of leaves	In vitro assay of radial growth in petri dish of *Colletotrichum kahawae*.	60% and 40% inhibition of growth in aqueous and ethanol extracts, respectively.	[35]
4	Anti-diabetes	Methanol extract of leaves and solvent fractions (water, ethyl acetate, and chloroform) 15.6–500 μg/mL	α-amylase inhibition using 3,5-dinitrosalicylic acid (DNSA) assays	74.52% (IC_50_, 14.52 μg/mL) followed by water fraction 68.24% (IC_50_, 16.31 μg/mL), ethyl acetate fraction 61.57% (IC_50_, 18.73 μg/mL), and chloroform fraction 56.87% (IC_50_, 21.57 μg/mL) of H. abyssinica leaves.	[5]
			α-glucosidase inhibiton of the plant extract were assessed p-nitro-phenyl-a-D glucopyranoside (p-NPG) assays	Aqueous fraction 62.54% (IC_50_, 11.67 μg/mL) followed by ethyl acetate fraction 54.97% (IC_50_, 15.89 μg/mL), crude extract 46.79% (IC_50_, >16.5 μg/mL), and chloroform fraction 36.44% (IC_50_, >16.5 μg/mL).	
		Methanol crude extract and solvent fractions (water, ethyl acetate, and chloroform) 25 to 800 μg/mL.	In vitro α-amylase inhibition assay was conducted through DNSA method	26.18 ± 0.88 (water) 28.27 ± 0.74 (chloroform) 43.38 ± 0.78 (crude extract) 54.23 ± 0.53 (ethyl acetate) 91.87 ± 0.54 (Acarbose).	[16]
5	Anticancer activity	Essential oil extracted from the female flowers of HA through hydo-distillation	Anti-proliferative assay on HL-60 Cell line	IC_50_: 50.07 μg/mL	[18]
		Methanol extract of HA roots and its fraction in water, n-hexane, dichloromethane, and ethyl acetate	MTT assay conducted on HepG2, cell line	^#^ IC_50_ values were 162, 30, and 41 for ethanol extract, n-hexane, and ethyl acetate fractions.	[2]
			MTT assay conducted on HT-29, cell line	^#^ IC_50_ values were 102, 50, and 59 for ethanol extract, n-hexane, and ethyl acetate fraction, respectively.	
			MTT assay conducted on SGC-7901 cell line	^#^ IC_50_ values were 137, 58, and 46 for ethanol extract, n-hexane, and ethyl acetate fractions.	
6	Anti-parasite	Essential oil extracted from the female flowers of HA through hydo-distillation	Antitrypanosomal activity against *Trypanosoma brucei*	IC_50_ = 42.30 μg/mL	[18]
		Ethanol crude extract of root and fractions in n-hexane, dichloromethane, ethyl acetate, and water.	Inhibition rates (IRs) of sample after cultured with *Trypanosoma brucei* for 24 h.	Crude extract 51% inhibition at low dose and 100% inhibition at high dose. Ethyl acetate fraction 100% inhibition at both low and high dose.	[21]
			Acetylcholinesterase inhibitory activity by Ellman’s method	IC_50_ values for ethyl acetate, crude extract, n-hexane, dichloromethane, and water fractions were 12.85 ± 1.82, 144.05 ± 20.58, 632.80 ± 1.00, 250.15 ± 20.44, and 211.50 ± 11.17 μg/mL, respectively.	
		Phytochemical isolated from the roots of HA	Inhibition of acetylcholinesterase through ultrafiltration-liquid chromatography–mass spectrometry based assay.	Binding degree % of phytochemicsals were 33.26, 49.81, 27.99, 33.74, 27.11, 40.34, and 26.57 for protocatechuic acid, corilagin, brevifolin carboxylic acid, brevifolin, methyl brevifolin carboxylate, quercetin, and methyl ellagic acid, respectively	
			Inhibition of glutathione reductase through ultrafiltration-liquid chromatography–mass spectrometry based assay.	Binding degree % of phytochemicsals were 15.55, 10.02, 18.43, 20.96, 1.32, and 17.26 for protocatechuic acid, brevifolin carboxylic acid, brevifolin, methyl brevifolin carboxylate, quercetin, and methyl ellagic acid, respectively.	
			Inhibition of lactate dehydrogenases through ultrafiltration-liquid chromatography–mass spectrometry based assay.	Binding degree % of phytochemicsals were 8.27, 4.94, 29.73, 8.17, and 12.16 for brevifolin carboxylic acid, brevifolin, methyl brevifolin carboxylate, quercetin, and methyl ellagic acid, respectively.	
		Female flowers of HA and its fraction in n-heptane, ethyl acetate, and methanol	100 μg/mL *Schistosoma mansoni*	Time of death were 3, 3, 3, and 166 h in crude extract, n-heptane, ethyl acetate, and methanol fractions. respectively.	[22]
			*Clonorchis sinensis*	Time of death were 5, 5, 5, and 8 h in crude extract, n-heptane, ethyl acetate, and methanol fractions. respectively.	
			*Fasciola hepatica*	Time of death were 51, 17, 41, and >72 h in crude extract, n-heptane, ethyl acetate, and methanol fractions. respectively.	
			*Echinostoma caproni*	Time of death were 1, 1, 18, and 1 h in crude extract, n-heptane, ethyl acetate, and methanol fractions. respectively.	

^#^ Approximate values derived from the graph provided in the article; DCM: dichloromethane; DNSA: 3,5-dinitrosalicylic acid; EC: *Escherichia coli*; MIC: minimum inhibition concentration; MIZ: mean inhibition zones; HA: *Hagenia abyssinica*; KP: *Klebsiella pneumoniae*; SA: *Staphylococcus aureus*; MRSA: Methicillin-resistant *Staphylococcus aureus*; SE: *Staphylococcus epidermidis*; ST: *Salmonella typhimurium*; SP: *Streptococcus pneumoniae*; PA: *Pseudomonas aeruginosa*; p-NPG: p-nitro-phenyl-a-D glucopyranoside; MG: *Microsporum gypseum*; TM: *Trichophyton mentagrophytes*; ABTS: 2,2′-azino-bis(3-ethylbenzothiazoline-6-sulfonic acid); DPPH: 1,1-diphenyl-2-picrylhydrazil; FRAP: Ferric-reducing ability power; MTT, 3-(4,5-dimethylthiazol-2-yl)-2,5-diphenyltetrazolium bromide.

**Table 3:** Pharmacological activities of HA studies in vivo ex vivo and human studies.

Sr. No.	Activity	Material	Method and Model	Results	Reference
1	Antidiarrheal	Fraction of leaves extract in aqueous, ethyl acetate, and chloroform and crude extract	Inhibition of defecation in castor oil-induced diarrhea in Swiss albino mice (Loperamide 3 mg/kg used as positive control)	84.60, 78.00, 57.91, 38.46, and 73.85% inhibtion of defecation in positive control, aqueous, ethyl acetate, chloroform, and crude extract groups respectivly.	[13,43]
			Castor oil-induced gastrointestinal motility in Swiss albino mice	59.80, 58.83, 51.92, 31.70, and 54.00% inhibtion of gastrointestinal motility in positive control, aqueous, ethyl acetate, chloroform, and crude extract groups respectivly.	
			Castor oil-induced enteropooling in Swiss albino mice	50.60, 47.00, 53.00, 40.70, and 46.00% inhibtion of enteropooling in positive control, aqueous, ethyl acetate, chloroform, and crude extract respectivly.	
			Antidiarrheal index (ADI) in Swiss albino mice	97.13, 74.14, 72.17, 52.37, and 81.24 in vivo ADI in positive control, aqueous, ethyl acetate, chloroform, and crude extract groups respectivly	
2	Anti-spasmodic activity	Water extract of the female flowers	Guinea pig ileum induce by the selected spamogens (acetyl choline, histamine, and barium chloride)	level of contraction ↓ amplitude of contraction of the guinea pig ileum ↓ induced by histamine.	[44]
3	Anticancer	α-kosin, kosotoxin, and protokosin isolated from the female flowers The single dose of 50 mg/kg and split doses of 12.5 mg/kg (for 4 days)	NMRI mice after transplantation of MAC tumors	Survival time ↑ in case of split doses of (protokosin and kosotoxin)	[45]
4	Anti-inflammatory activity	Methanol extract fraction in ethyl acetate, chloroform and water; Oral doses: 100, 200, and 400 mg/kg	Carrageenan-induced hind paw edema model in mice. Indomethacin (positive control)	Paw edema ↓ (in all 3 doses)	[6]
5	Wound-healing	Methanol (80%) extract of flowers and fractions in water, ethyl acetate and chloroform used to prepared ointment (5 and 10% *w*/*w*)	Swiss albino mice excision wound model	Time of contraction of wound ↓ and epithelization of period ↓	
		Methanol (80%) extract of flowers (5 and 10% *w*/*w*)	Swiss albino mice incision wound model	Tensile strength ↑ (In both doses)	
6	Anti-diabetes	Doses: 100, 200, and 400 mg/kg of crude extract of flower	Hypoglycemic activity at baseline, 1, 2, 4, and 6 h in healthy male Swiss albino mice (Normoglycemic mice model)	BGL ↓ (in 200 and 400 mg/kg groups on the 6th hour after the administration of extracts)	[16]
			Oral glucose tolerance test on healthy male Swiss albino mice oral administration of glucose (2 g/kg) after 30 min of extract administration.	BGL ↓ (in 200 and 400 mg/kg groups after 120 min of the oral administration of glucose)	
		Doses: 100, 200, and 400 mg/kg of crude methanol extract of flower and its fraction ethyl acetate.	Anti-hyperglycemic activity in STZ-induced male Swiss albino mice for a single dose of extracts.	BGL ↓ (at the 8th hour after treatment of 400 mg/kg crude extract and ethyl acetate fraction)	
			Anti-hyperglycemic activity in STZ-induced male Swiss albino mice for daily doses of extracts.	BGL ↓ (at 7th and 14th days after the start of daily treatment of 200 and 400 mg/kg crude extract and ethyl acetate fraction)	
			Body weight in STZ-induced male Swiss albino mice for daily doses of extracts.	BW ↑ (at the 7th day after the start of daily treatment of 200 and 400 mg/kg doses of crude extract and 400 mg/kg dose of ethyl acetate); BW ↑ (on the 14th day all doses of crude and ethyl acetate fraction)	
			Serum lipid profile in STZ-induced male Swiss albino mice for daily doses of extracts.	TC ↓, TG ↓, LDL ↓, and VLDL ↓ (all doses of crude extract and 400 mg/kg ethyl acetate fraction groups);HDL-c ↑ (all doses of crude extract and 200 and 400 mg/kg of ethyl acetated fractions)	
		Doses: 100, 200, and 400 mg/kg of crude methanol extract of leaves (100, 200, and 400 mg/kg)	Anti-hyperglycemic activity in STZ-induced male Swiss albino mice for daily doses of extracts.	BGL ↓ (at 7th and 14th days after the start of daily treatment of all doses).	[23]
			Body weight in STZ-induced male Swiss albino mice for daily doses of extracts.	BW ↑ (7th day after the start of daily treatment of 400 mg/kg dose) BW ↑ (on the 14th day in 200 and 400 mg/kg doses groups)	
			Serum lipidprofiles in STZ-induced male Swiss albino mice for daily doses of extracts.	Serum levels of TC ↓, TG ↓, VLDL-c ↓, LDL-c ↓, and HDL-c ↑ (15th day after the start of daily treatment on all doses).	
7	Anti-parasite activity	Dried HA material at 20, 40, and 60 mg doses	Cestode egg count in feces of Alpine goats.	Egg count ↓ (in all 3 doses)	[42]
		Powdered plant material (flowers) with honey one time in the morning	Group of 6 humans (worm-infested subjects) in each dose group	MESD was 12.5 ± 2.2 g and WET was 11.3 ± 1.4 (hour)	[14]

↑: up-regulation; ↓: down-regulation/inhibition; BGL: blood glucose level; BW: body weight; MESD: Median effective single dose; WET: worm expulsion time; TC: cholesterol; TG: triglyceride; HDL-c: high-density lipoprotein cholesterol; VLDL-c: very-low-density lipoprotein cholesterol; LDL-c: low-density lipoprotein cholesterol; STZ: streptozotocin.

## Data Availability

Data are contained within the article.
